# Healthcare Professional's Perception of Patient Safety Measured by the Hospital Survey on Patient Safety Culture: A Systematic Review and Meta-Analysis

**DOI:** 10.1155/2018/9156301

**Published:** 2018-07-19

**Authors:** Julia Hiromi Hori Okuyama, Tais Freire Galvao, Marcus Tolentino Silva

**Affiliations:** ^1^Universidade de Sorocaba, Graduate Program of Pharmaceutical Science, Sorocaba, Brazil; ^2^Universidade Estadual de Campinas, Faculty of Pharmaceutical Sciences, Campinas, Brazil

## Abstract

**Objective:**

To assess the culture of patient safety in studies that employed the hospital survey on patient safety culture (HSOPS) in hospitals around the world.

**Method:**

We searched MEDLINE, EMBASE, SCOPUS, CINAHL, and SciELO. Two researchers selected studies and extracted the following data: year of publication, country, percentage of physicians and nurses, sample size, and results for the 12 HSOPS dimensions. For each dimension, a random effects meta-analysis with double-arcsine transformation was performed, as well as meta-regressions to investigate heterogeneity, and tests for publication bias.

**Results:**

59 studies with 755,415 practitioners surveyed were included in the review. 29 studies were conducted in the Asian continent and 11 in the United States. On average studies scored 9 out of 10 methodological quality score. Of the 12 HSOPS dimensions, six scored under 50% of positivity, with “nonpunitive response to errors” the lowest one. In the meta-regression, three dimensions were shown to be influenced by the proportion of physicians and five by the continent where survey was held.

**Conclusions:**

The HSOPS is widely used in several countries to assess the culture of patient safety in hospital settings. The culture of culpability is the main weakness across studies. Encouraging event reporting and learning from errors should be priorities in hospitals worldwide.

## 1. Introduction

Health institutions, which are known to be complex organizations, have over the years devised improvement strategies and added quality to the health care service [1]. Patient's safety culture reflects the perceptions of processes, norms, and attitudes relating to a culture of preventable errors shared by health professionals in the delivery of care [2]. In health environments, behaviors and attitudes shape the culture of each organization [3]. The sharing of beliefs, values, and attitudes related to the patient's safety culture influences the outcome and organizational aspects [4].

Higher culture of patient safety has been shown to be associated with better patient outcomes [5]. Quality in hospital services means providing the patient with multidisciplinary care at minimal risk [6]. Therefore, implementing improvements in organizational and safety culture enhances quality [7].

Surveys are widely used to assess the culture of safety by identifying the perception of health practitioners [8]. Such inquiries allow for a general assessment of the work climate, the relationship between teams or in a given group, communications, professional relationships, and hierarchical relations. They can identify areas that need prioritization for interventions.

Among the validated tools [8], the Hospital Survey on Patient Safety Culture (HSOPS) and the Safety Attitudes Questionnaire (SAQ), both created in the United States of America (US), are widely cited in research that aims to assess the safety culture of patients in different countries [9]. The HSOPS was designed by the US Agency of Healthcare Research and Quality in 2004 and proposes the assessment of 12 dimensions pertaining to the climate of patient safety in hospital setting. Seven dimensions of the survey are related to the work area, three dimensions explore aspects of the safety culture in the hospital, and two are outcome variables [10]. The culture of safety is measured by the staff perspective. For each dimension, percentages above 75% are considered as strengths and below 50% are areas that need improvement [10].

Since its inception, HSOPS has been translated and validated in several languages and settings [11–17]. To date, no compilation of surveys that employed HSOPS in hospitals in different countries. This comparison would bring valuable sources of strengths and limitations of culture of patient safety.

The aim of present study is to summarize surveys that assessed the culture of patient safety by HSOPS in hospitals worldwide, by means of a systematic review and meta-analysis.

## 2. Methods

### 2.1. Protocol and Registration

The protocol for this review was prepared in advance and registered in the International Prospective Register of Systematic Reviews (PROSPERO) under registration number CRD42016047941.

### 2.2. Eligibility Criteria

Studies meeting the criteria were selected as follows: Portuguese, Spanish, or English language; publication date between 2008 and 2015; cross-sectional, hospital-based design; full or partial use of the HSOPS questionnaire; inclusion of staff with direct or indirect patient contact; and surveying one or more categories of health professionals.

Studies were excluded if they were performed outside of the hospital setting, if they were conducted in a single area or unit of the hospital, or if they were lacking in results for each dimension. Additionally, validation studies, duplicate studies, papers with no full-text available, or studies that used qualitative approaches were excluded.

### 2.3. Information Sources and Search Strategy

The following databases were searched: MEDLINE (via PubMed), EMBASE, SCOPUS, CINAHL, and SciELO.

The search terms used for PubMed that were adapted for the other databases were “HSOPSC OR (Hospital Survey on Patient Safety Culture)”.

### 2.4. Study Selection

The Covidence software platform (www.covidence.org) was used to organize the references and find duplicates. Two independent reviewers screened the titles and abstracts of the papers, with selected papers progressing to the second phase, which was a full-text review. The articles were evaluated according to pre-specified criteria and, in the event of disagreements in either of the two phases, a third reviewer determined the inclusion.

### 2.5. Data Collection Process

Two reviewers independently summarized the data using a data extraction sheet. The following information was collected from each paper: year, country, proportion of physicians and nurses, sample size, and results for the 12 HSOPS dimensions (Table 1).

### 2.6. Risk of Bias

Two reviewers evaluated the studies independently using a validated 10-item tool, which assessed (i) representativeness of the sample, (ii) appropriateness of the recruitment, (iii) adequacy of the sample size, (iv) description of both the study subjects and the setting, (v) response rate, (vi) objective, standard criteria used for the measurement of the condition, (vii) reliability of the measurement of the condition, (viii) appropriateness of the statistical analysis, (ix) important confounding factors/subgroups/differences that were identified and accounted for, and (x) subpopulations that were identified using objective criteria [18]. Disagreements were resolved by consensus.

### 2.7. Summary Measures and Statistical Analysis

The outcome measure for this study was the proportion of positive responses in each dimension. STATA statistical software V.14.2 was used for all calculations.

For each HSOPS dimension, meta-analyses were performed by grouping the positive scores using the random effects model described by DerSimonian and Laird and the double-arcsine transformation for variance stabilization as proposed by Freeman-Tukey [19, 20]. The prediction confidence interval was also calculated [20, 21].

Heterogeneity was assessed by calculating the inverse variance in a fixed-effects model, which was expressed as a percentage of the* I*^2^ statistic [20, 22]. Among the study characteristics, possible causes of heterogeneity were investigated: year of publication, proportion of physicians, proportion of nurses, quality scores, and continent where the study was performed. A meta-regression was performed of the double-arcsine transformed results in the method-of-moments model with a restricted maximum likelihood and a modified coefficient variance as suggested by Knapp and Hartung [23, 24]. Thus, the *β* coefficient, the probability (*p* value), and the residual heterogeneity were calculated. Values of* p* < 0.05 were deemed significant.

Publication bias (small study effect) was investigated using three approaches for each dimension. The first consisted of a regression of the log odds of the positive results against their standard errors (Egger's test). The second strategy was a regression of the odds against the reciprocal of the sample size (Peters' test). For both tests, probability values below 0.10 were deemed significant [25]. The third approach included a visual assessment of asymmetry in two funnel plots: one that compared sample size against log odds and the other that compared the log odds against the standard errors [25, 26].

## 3. Results

**Figure 1:**
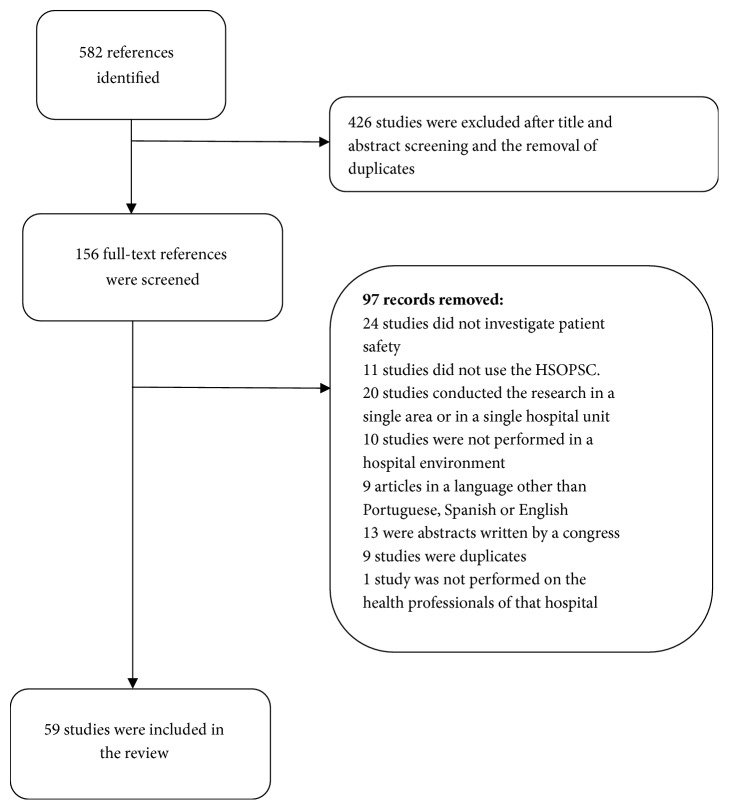
Flowchart of included studies.

The search retrieved 582 studies, of which 59 were included [5, 27–83] (Figure 1).

Eleven studies were published prior to 2011; and 48, between 2011 and 2015 (Table 2). Twenty-nine were conducted in Asia (of note, eight in Iran), 18 occurred in Europe, and 14 occurred in American continent, of which 11 were in the US. Two studies were located in Eurasia (Turkey) and one was in Africa (Egypt).

The studies included 755,415 professionals who answered the HSOPS, of which 55.4% were nurses and 5.2% were physicians. Sample sizes ranged from 90 to 247,140 participants (Table 2). The largest studies were conducted in the US [63, 64, 78] while the smallest studies were Iranian[41].

One study surveyed the same institution three times on different years [37]. Thirty-three studies included a variety of professionals, 22 surveyed nursing staff exclusively and one surveyed only physicians [73]. Four investigations were multi-center studies [31, 35, 51, 66] (surveys administered at different hospitals in the same country) and three were international multi-center surveys [54, 63, 64].

The mean score for methodological quality of the evaluated articles, on a scale of 0 to 10, was 9.0 points, with 34 studies that achieved the maximum score (Table 2). Considering the appraisal criteria, 19 studies showed errors in the participant recruitment process, 12 neglected the calculation of sample size, and nine failed to report response rates.

The results of the meta-analyses of the 12 HSOPS dimensions are presented in Table 3. Five had less than 50% of positivity in the dimensions of “communication openness”, “frequency of events reported”, “staffing”, “handoffs and transitions”, and “nonpunitive response to errors”. Only the dimension of “teamwork within units” produced positive responses in 75% of those surveyed, which was the highest percentage.

A survey that was conducted in Norway in 2012 produced positive responses in 78.8% of the “nonpunitive response to errors” dimension,[42] and an analysis performed in Spain, reported 3.7% positivity for the dimension of “management support for patient safety”[39], while seven others had positive scores of less than 30% [28, 36, 40–42, 55, 72].

The meta-analyses detected high heterogeneity values across the HSOPS dimensions, with all that were above 97%. The meta-regression showed that three dimensions were influenced by the proportion of doctors in the dimensions of “overall perceptions of patient safety”, “feedback and communication about error”, and the “frequency of events reported” (Table 4). The continent where the survey was held significantly affected the dimensions of “supervisor/manager expectations and actions promoting patient safety” (America 72-76%* versus* others 42-67%), “overall perceptions of patient safety” (America 58-67%* versus* others 30-55%), “communication openness” (America 59-63%* versus* others 31-63%), “staffing” (America 47-64%* versus* others 31-63%), and “nonpunitive response to errors” Asia and Eurasian 23-32%* versus* Europa and America 36-58%). Only the dimensions of “organizational learning-continuous improvement”, “teamwork within units”, and “handoffs and transitions” were not positive for small study effects. Funnel plot inspections showed asymmetry in all dimensions (data not shown).

## 4. Discussion

The present review made it possible to identify studies that used HSOPS and to evaluate the safety culture of patient in hospital setting worldwide. There are still aspects in the safety culture of patient that deserve attention to improve patient care in these environments. HSOPS was used both in a specific class of professionals as well as to all hospital staff.

The dimension “nonpunitive response to errors” was the one with the lowest score and “teamwork among the units” the highest score regarding the patient safety items addressed in the survey. Similar results were found in a systematic review and meta-analysis of the HSOPS conducted to assess the patient's safety culture in hospitals in Iran [84]. This review included Iranian surveys conducted between 2000 and 2014 and used a writing tool as an instrument of critical evaluation, with calculation of meta-analysis using simple means of the domains.

The weakest dimensions were those that were related to communication problems and staffing, with the “nonpunitive response to error” the worst rated dimension. This may reflect the culpability culture of the hospitals but also a comprehensiveness problem. This domain has only negative questions, which induces misunderstanding and less reliability in questionnaires [85]. Dimensions with lower scores may reflect the wording and not the limitation in safety culture. External analyses of HSOPS [86] showed possible weaknesses in its psychometric properties.

Evaluating perceptions of the culture of safety implies the consideration of a number of factors and characteristics pertaining to the hospital setting [87]. Management that is committed to safety culture, effective leadership support, effective communication, sufficient staffing, incentives to capacity-building, and interdisciplinary teamwork are just some of those factors [72]. Unities with different profiles in terms of their constitution and organization (specialized intensive care units, emergency departments, surgical suites, and wards) are found in hospitals [88]. In those unities, perceptions of patient safety vary between practitioners [32, 42]. In this review, the influence of the medical staff was noted for some dimensions. Thus, to assess the perception of patient safety through surveys, the influence of the context of each unit and different professionals should also be considered.

Studies that evaluated the culture of safety have shown contrasting perceptions regarding patient safety in different professionals, and in one study, physicians showed a less positive perception compared to nurses [89] and more positive perceptions compared to the nursing staff in another [90, 91].

The continent where the study was conducted was a source of variability of results across studies. In international multi-center studies [54, 63], a greater proportion of positive scores was found in the US than elsewhere. In addition to the cultural differences [63], the HSOPS was developed in the US; hence its use is more disseminated in that country, a fact that becomes evident in the sample sizes. Another factor detected in those multi-center studies is the larger number of nurses in US hospitals compared to other countries [54], a fact that is attributable to the way hospitals adapt the numbers of nurses to their demand and hire temporary staff.

In a Norwegian study [42], a salient strength was found in the “nonpunitive response to error” dimension. Another study conducted in the same country [92] found a similar value for this dimension. In most studies that were conducted in a variety of countries, this dimension yielded low scores, which indicated the need for improvement. Other investigations were pursued [28, 32, 55, 58, 60, 63, 70] with higher positive percentages in this dimension and seven were found with positive scores above 50%. It was noted that, in the settings where these surveys were administered, a climate of encouragement existed in the management staff who promoted reporting and learning from errors.

Included studies showed good methodological quality, which showed no effect on the heterogeneity, partly attributable to the proportion of physicians and the location where the surveys were administered. The statistical tests indicated that small study effect was present and publication bias may have influenced the results. Studies published in scientific conferences and in other gray literature were not included and the restriction of only publications in Portuguese, English, and Spanish may have stressed out this effect. With the purpose of extending the usability and determining the applicability of the HSOPS, all sample sizes and professional categories were included, which influenced the high heterogeneity.

As a psychometric tool, HSOPS adaptation versions [93] are subject to inconsistences due to language and cultural particularities. A previous validation of the tool was not an eligibility criterion in our review. This may have overestimated or underestimated our findings and probably contributed to the high heterogeneity. HSOPS showed good psychometric properties of safety culture [10] when assessed by its development group. Like other questionnaires, the link between the society culture and patient safety may be an issue and could be better explored by qualitative assessment.

The methods of this review were based on internationally recommended standards [94]. Thus, paired reviewers worked on the inclusion, evaluation, and data extraction steps. Data analysis relied on statistical calculations grouped according to relevant variables. The use of the HSOPS is still emerging in some countries. In accordance with the worldwide trend towards patient safety in the health services, this systematic review could foster the use and dissemination of the HSOPS.

## 5. Conclusions

The culture of culpability is pervasive in most of the hospitals that measured the culture of safety using the HSOPS. This behavior reduces error reporting and the likelihood that corrective measures would be implemented. Effective communication, feedback following reporting, engaged leadership, and environments focused on learning from errors are factors that can lead to improvement.

## Figures and Tables

**Table 1:** Hospital Survey on Patient Safety Culture dimensions and what they are intended to measure.

**Dimensions of patient safety culture related to the work area or unit**	
(1) Teamwork within units	Supervisors and managers consider employee suggestion about patient safety, teamwork and open communication about errors, work hours are adequate to provide the best patient care. Feedback from management, and continuous improvement to avoid errors.
(2) Supervisor/manager expectations and actions that promote patient safety	
(3) Organizational learning and continuous improvement	
(4) Communication openness	
(5) Feedback and communication about error	
(6) Staffing	
(7) Nonpunitive response to errors	
**Dimensions explore aspects of the safety culture in hospital**	
(8) Management support for patient safety	Hospital management support patient safety and patient care information are not lost during shift change and from one unit to another
(9) Teamwork across units	
(10) Handoffs and Transition	
**Dimensions of outcome variables**	
(11) Overall perceptions of patient safety	Existence of procedures to avoid the occurrence of errors, notifications of possible problems and corrections before they affect the patient
(12) Frequency of events reported	

**Table 2:** Characteristics of included studies (n=59).

Continent	Countries	Author, year	Sample size	Proportion of physicians	Proportion of nurses	Quality score
Africa	Egypt	Aboul-Fotouh, 2012	510	50.0	32.4	10
America	Brazil	Silva-Batalha, 2015	301	-	18.9	9
	Colombia	Gómez Ramírez, 2011	201	-	54.7	10
	United States of America	Blegen, 2010^a^	368	40.0	33.0	9
			434	34.0	30.0	
		Bump, 2015	955	100.0	-	7
		Campbell, 2010	2,163	19.9	80.1	10
		Dupree, 2011^b^	163	25.0	37.0	10
			234	21.0	48.0	
			325	-	-	
		Halbesleben, 2008	148	-	100.0	10
		Jones, 2013	2,137	9.4	32.0	10
		Mardon, 2010	179	-	-	10
		Patterson, 2015	247,140	4.7	51.2	10
		Ulrich, 2014	979	-	100.0	10
		Wagner, 2013^c^	196,462	4.0	36.0	8
		Wu, 2013^d^	106,710	-	100.0	9
	Mexico	Castañeda-Hidalgo, 2013	195	-	90.3	10
Asia	Saudi Arabia	Aboshaiqah, 2010	445	-	100.0	10
		Aboshaiqah, 2013	498	-	100.0	10
		Alahmadi, 2010	1,224	8.3	60.0	9
		Al-Ahmadi, 2009	1,224	8.8	63.7	9
		Al-Awa, 2012	605	-	100.0	7
		El-Jardali, 2014	2,572	8.7	50.1	10
	China	Nie, 2013	1,160	25.9	62.2	8
		Shu, 2015	2,230	31.0	69.0	10
		Wang, 2014	463	-	100.0	10
	Iran	Adibi, 2012	90	7.8	71.1	3
		Al-Mandhari, 2014	398	20.9	59.5	10
		Ammouri, 2015	414	-	100.0	10
		Bahrami, 2013^e^	135	-	100.0	9
			135	-	100.0	
		Bahrami, 2014^f^	113	-	100.0	10
			189	-	100.0	
		Davoodi, 2013	922	10.0	77.0	10
		Moussavi, 2013	175	32.6	41.7	10
		Raeissi, 2015	461	15.2	51.0	10
	Japan	Fujita, 2013^h^	6,963	8.5	58.1	10
		Fujita, 2014	8,700	9.3	46.4	9
		Wu, 2013^d^	4,047	-	100.0	9
	Jordan	Khater, 2015	658	-	100.0	10
		Saleh, 2015	242	-	100.0	10
	Lebanon	El-Jardali, 2010	6,807	3.7	57.8	10
	Palestine	Hamdan, 2013	1,408	20.0	49.2	10
	Taiwan	Chen, 2012	788	29.2	60.7	10
		Chen, 2010	788	29.2	60.7	10
		Fujita, 2013^h^	10,019	9.7	57.0	10
		Wagner, 2013^c^	10,146	10.0	58.0	8
		Wu, 2013^d^	5,714	-	100.0	9
Eurasian	Turkey	Günes, 2015	554	-	100.0	8
		Ugurluoglu, 2012	108	27.8	42.6	9
Europe	Belgium	Vlayen, 2012	55,225	8.8	49.8	10
		Hellings, 2010^g^	3,626	12.2	60.5	10
			3,940	11.7	64.1	
		Vlayen, 2015	47,136	9.6	52.5	9
	Croatia	Brborovic, 2014	148	-	100.0	8
		Sklebar, 2013	560	-	-	5
	Scotland	Agnew, 2013	1,866	-	53.0	5
	Western Slovakia	Mikusová, 2012	1,787	13.6	50.5	9
	Spain	Gama, 2013	1,113	24.7	45.0	10
		Saturno, 2008	2,503	-	-	8
		Skodova, 2011	299	-	40.1	10
	Finland	Kuosmanen, 2013	283	6.4	82.2	6
		Turunen, 2013	832	-	100.0	6
	Italy	Bagnasco, 2011	724	35.0	26.0	7
	Norway	Ballangrud, 2012	220	-	100.0	10
		Farup, 2015	185	14.1	61.6	10
	Netherlands	Smits, 2012	542	16.5	74.0	6
		Wagner, 2013^c^	3,779	12.0	53.0	8
	United Kingdom	Lawton, 2015	648	-	100.0	8

*Note.* ^a,b,g^ Different years: ^a^2006 and 2007, ^b^2005, 2008, and 2011, and ^g^2005 and 2007; ^c,d^same study conducted on different continents; ^e,f^same study performed in different hospitals; ^h^same study conducted on different countries.

**Table 3:** Meta-analyses of the dimensions and respective heterogeneity (I^2^) of the Hospital Survey on Patient Safety Culture (n=59 studies).

Dimensions	Positive responses, % (95% CI)	I^2^ (%)
Teamwork within units	75 (73-76)	97.9
Supervisor/Manager expectations and actions that promote patient safety	61 (59-64)	99.0
Organizational learning and continuous improvement	70 (67-73)	99.3
Management support for patient safety	53 (48-57)	99.6
Overall perceptions of patient safety	54 (51-56)	98.9
Feedback and communication about error	54 (51-57)	99.1
Communication openness	47 (44-51)	99.4
Frequency of events reported	48 (45-52)	99.5
Teamwork across units	50 (47-53)	98.9
Staffing	36 (33-40)	99.5
Handoffs and transitions	45 (44-47)	97.7
Nonpunitive response to errors	33 (30-37)	99.4

*Note.* Positive responses in percentage. 95% CI, 95% predictive confidence interval.

**Table 4:** Investigation of causes of heterogeneity by meta-regressions and testing for publication bias by Egger's and Peters' tests of each Hospital Survey on Patient Safety Culture dimension (n=59 studies).

Dimensions	Double arcsine coefficient (*β*), p value, residual heterogeneity (I^2^, %) of meta-regression															Publication bias	
	Year			Doctors			Nurses			Quality score			Continent			*p *value	
	*β*	*p* value	I^2^	*β*	*p* value	I^2^	*β*	*p* value	I^2^	*β*	*p* value	I^2^	*β*	*p* value	I^2^	Egger's test	Peters' test
Teamwork within units	0.013	0.424	97.9	-0.006	0.166	98.5	<0.001	0.808	98.0	0.008	0.416	97.8	-0.029	0.458	97.8	0.071	0.005
Supervisor/Manager expectations and actions that promote patient safety	-0.031	0.125	99.0	-0.005	0.154	98.9	-0.005	0.100	98.1	0.001	0.926	98.2	-0.139	<0.001	95.8	0.001	<0.001
Organizational learning and continuous improvement	0.018	0.374	99.3	<0.001	0.936	99.5	<0.001	0.224	99.3	0.001	0.953	99.2	0.068	0.146	99.3	0.980	0.490
Management support for patient safety	<0.001	0.987	99.6	-0.012	0.064	99.6	-0.012	0.307	99.6	0.002	0.903	99.6	-0.019	0.776	99.5	0.022	0.016
Overall perceptions of patient safety	-0.012	0.481	98.9	-0.007	0.039	98.7	-0.007	0.840	98.4	0.004	0.687	98.7	-0.078	0.018	97.9	0.021	0.016
Feedback and communication about error	0.008	0.694	99.2	-0.007	0.043	99.2	-0.007	0.504	98.9	0.011	0.329	99.1	-0.026	0.544	98.6	0.090	0.077
Communication openness	-0.019	0.390	99.4	-0.005	0.177	99.5	-0.005	0.739	99.1	-0.001	0.913	99.3	-0.131	0.002	98.0	0.017	0.023
Frequency of events reported	0.003	0.892	99.5	-0.009	0.049	99.7	-0.009	0.606	99.5	0.022	0.072	99.5	-0.070	0.145	99.4	0.074	0.089
Teamwork across units	0.013	0.526	98.9	-0.004	0.385	99.2	-0.004	0.467	98.9	0.012	0.270	98.7	-0.027	0.539	97.7	0.114	0.115
Staffing	-0.016	0.544	99.5	-0.003	0.570	99.5	-0.003	0.460	99.2	0.001	0.923	99.3	-0.132	0.012	98.3	0.005	0.008
Handoffs and transitions	0.010	0.562	97.5	-0.006	0.053	98.0	-0.006	0.273	97.7	0.003	0.755	97.7	-0.031	0.422	97.6	0.503	0.372
Nonpunitive response to errors	-0.041	0.159	99.4	-0.002	0.671	99.6	-0.002	0.771	99.3	0.009	0.577	99.4	-0.198	<0.001	99.0	0.044	0.111

## Data Availability

The data used to support the findings of this study are available upon request from the corresponding author.

## References

[1] Chassin M. R., Loeb J. M. (2013). High-reliability health care: Getting there from here. *Milbank Quarterly*.

[2] Zohar D., Livne Y., Tenne-Gazit O., Admi H., Donchin Y. (2007). Healthcare climate: A framework for measuring and improving patient safety. *Critical Care Medicine*.

[3] Kaufman G., McCaughan D. (2013). The effect of organisational culture on patient safety. *Nursing Standard*.

[4] Weaver S. J., Lubomksi L. H., Wilson R. F., Pfoh E. R., Martinez K. A., Dy S. M. (2013). Promoting a culture of safety as a patient safety strategy: A systematic review. *Annals of Internal Medicine*.

[5] Mardon R. E., Khanna K., Sorra J., Dyer N., Famolaro T. (2010). Exploring relationships between hospital patient safety culture and adverse events. *Journal of Patient Safety*.

[6] WHO (2005). *World Alliance for Patient Safety: forward programme*.

[7] Dodek P. M., Wong H., Heyland D. K. (2012). The relationship between organizational culture and family satisfaction in critical care. *Critical Care Medicine*.

[8] Colla J. B., Bracken A. C., Kinney L. M., Weeks W. B. (2005). Measuring patient safety climate: A review of surveys. *Quality & Safety in Health Care*.

[9] Kristensen S., Bartels P. (2010). Use of Patient Safety Culture Instruments and Recommendations. *European Society for Quality in Healthcare. Office for Quality Indicators*.

[10] Sorra J. S. N. V. (2004). *Hospital Survey on Patient Safety Culture. (Prepared by Westat, under Contract No. 290-96-0004). AHRQ Publication no 04-0041*.

[11] Eiras M., Escoval A., Grillo I. M., Silva-Fortes C. (2014). The hospital survey on patient safety culture in Portuguese hospitals: Instrument validity and reliability. *International Journal of Health Care Quality Assurance*.

[12] Bodur S., Filiz E. (2009). A survey on patient safety culture in primary healthcare services in Turkey. *International Journal for Quality in Health Care*.

[13] Brborović H., Šklebar I., Brborović O., Brumen V., Mustajbegović J. (2014). Development of a Croatian version of the US hospital survey on patient safety culture questionnaire: Dimensionality and psychometric properties. *Postgraduate Medical Journal*.

[14] Ito S., Seto K., Kigawa M., Fujita S., Hasagawa T., Hasegawa T. (2011). Development and applicability of Hospital Survey on Patient Safety Culture (HSOPS) in Japan. *BMC Health Services Research*.

[15] Waterson P., Griffiths P., Stride C., Murphy J., Hignett S. (2010). Psychometric properties of the Hospital Survey on Patient Safety Culture: Findings from the UK. *Quality & Safety in Health Care*.

[16] Vlayen A., Hellings J., Claes N., Abdou A., Schrooten W. (2015). Measuring Safety Culture in Belgian Psychiatric Hospitals: Validation of the Dutch and French Translations of the Hospital Survey on Patient Safety Culture. *Journal of Psychiatric Practice*.

[17] Reis C. T., Laguardia J., Vasconcelos A. G., Martins M. (2016). Reliability and validity of the Brazilian version of the Hospital Survey on Patient Safety Culture (HSOPSC): a pilot study. *Cadernos de Saúde Pública*.

[18] Munn Z., Moola S., Riitano D., Lisy K. (2014). The development of a critical appraisal tool for use in systematic reviews addressing questions of prevalence. *International Journal of Health Policy and Management*.

[19] Barendregt J. J., Doi S. A., Lee Y. Y., Norman R. E., Vos T. (2013). Meta-analysis of prevalence. *Journal of Epidemiology and Community Health*.

[20] Nyaga V. N., Arbyn M., Aerts M. (2014). Metaprop: A Stata command to perform meta-analysis of binomial data. *Archives of Public Health*.

[21] Turner R. M., Davey J., Clarke M. J., Thompson S. G., Higgins J. P. (2012). Predicting the extent of heterogeneity in meta-analysis, using empirical data from the Cochrane Database of Systematic Reviews. *International Journal of Epidemiology*.

[22] Higgins J. P. T., Thompson S. G. (2002). Quantifying heterogeneity in a meta-analysis. *Statistics in Medicine*.

[23] Knapp G., Hartung J. (2003). Improved tests for a random effects meta-regression with a single covariate. *Statistics in Medicine*.

[24] Harbord R. M., Higgins J. P. T. (2008). Meta-regression in Stata. *Stata Journal*.

[25] Jin Z.-C., Zhou X.-H., He J. (2015). Statistical methods for dealing with publication bias in meta-analysis. *Statistics in Medicine*.

[26] Hunter J. P., Saratzis A., Sutton A. J., Boucher R. H., Sayers R. D., Bown M. J. (2014). In meta-analyses of proportion studies, funnel plots were found to be an inaccurate method of assessing publication bias. *Journal of Clinical Epidemiology*.

[27] Halbesleben J. R. B., Wakefield B. J., Wakefield D. S., Cooper L. B. (2008). Nurse burnout and patient safety outcomes: Nurse safety perception versus reporting behavior. *Western Journal of Nursing Research*.

[28] Saturno P. J., Da Silva Gama Z. A., de Oliveira-Sousa S. L. (2008). Analysis of the patient safety culture in hospitals of the Spanish National Health System. *Medicina Clínica*.

[29] Al-Ahmadi T. A. (2009). Measuring Patient Safety Culture in Riyadh's Hospitals: A Comparison between Public and Private Hospitals. *Journal of Egyptian Public Health Association*.

[30] Alahmadi H. A. (2010). Assessment of patient safety culture in Saudi Arabian hospitals. *Quality & Safety in Health Care*.

[31] Blegen M. A., Sehgal N. L., Alldredge B. K., Gearhart S., Auerbach A. A., Wachter R. M. (2010). Republished paper: Improving safety culture on adult medical units through multidisciplinary teamwork and communication interventions: The TOPS project. *Postgraduate Medical Journal*.

[32] Campbell E. G., Singer S., Kitch B. T., Iezzon L. I., Meyer G. S. (2010). Patient safety climate in hospitals: Act locally on variation across units. *Joint Commission Journal on Quality and Patient Safety*.

[33] Chen I.-C., Li H.-H. (2010). Measuring patient safety culture in Taiwan using the Hospital Survey on Patient Safety Culture (HSOPSC). *BMC Health Services Research*.

[34] El-Jardali F., Jaafar M., Dimassi H., Jamal D., Hamdan R. (2010). The current state of patient safety culture in lebanese hospitals: A study at baseline. *International Journal for Quality in Health Care*.

[35] Hellings J., Schrooten W., Klazinga N. S., Vleugels A. (2010). Improving patient safety culture. *International Journal of Health Care Quality Assurance*.

[36] Bagnasco A., Tibaldi L., Chirone P. (2011). Patient safety culture: An Italian experience. *Journal of Clinical Nursing*.

[37] Dupree E., Anderson R., McEvoy M. D., Brodman M. (2011). Professionalism: A necessary ingredient in a culture of safety. *Joint Commission Journal on Quality and Patient Safety*.

[38] Gómez Ramírez O., Arenas Gutiérrez W., González Vega L., Garzón Salamanca J., Mateus Galeano E., Soto Gámez A. (2011). Cultura de seguridad del paciente por personal de enfermería en bogotá, Colombia. *Ciencia y enfermería*.

[39] Skodova M., Velasco Rodriguez M. J., Fernandez Sierra M. A. (2011). Opinion of healthcare professionals on patient safety in a primary level hospital. *Revista de Calidad Asistencial*.

[40] Aboul-Fotouh A. M., Ismail N. A., Ez Elarab H. S., Wassif G. O. (2012). Assessment of patient safety culture among health-care providers at a teaching hospital in Cairo, Egypt. *Eastern Mediterranean Health Journal*.

[41] Adibi H., Khalesi N., Ravaghi H., Jafari M., Jeddian A. (2012). Development of an effective risk management system in a teaching hospital. *Journal of Diabetes & Metabolic Disorders*.

[42] Ballangrud R., Hedelin B., Hall-Lord M. L. (2012). Nurses' perceptions of patient safety climate in intensive care units: A cross-sectional study. *Intensive and Critical Care Nursing*.

[43] Al-Awa B., Al Mazrooa A., Rayes O. (2012). Benchmarking the post-accreditation patient safety culture at King Abdulaziz University Hospital. *Annals of Saudi Medicine*.

[44] Chen I.-C., Ng H.-F., Li H.-H. (2012). A multilevel model of patient safety culture: Cross-level relationship between organizational culture and patient safety behavior in Taiwan's hospitals. *International Journal of Health Planning and Management*.

[45] Mikušová V., Rusnáková V., Naďová K., Boroňová J., Beťková M. (2012). Patient safety assessment in Slovak hospitals. *International Journal of Collaborative Research on Internal Medicine and Public Health*.

[46] Smits M., Wagner C., Spreeuwenberg P., Timmermans D. R. M., van der Wal G., Groenewegen P. P. (2012). The role of patient safety culture in the causation of unintended events in hospitals. *Journal of Clinical Nursing*.

[47] Ugurluoglu O., Ugurluoglu E., Payziner P. D., Ozatkan Y. (2012). Patient safety culture: Sample of a University Hospital in Turkey. *Pakistan Journal of Medical Sciences*.

[48] Vlayen A., Hellings J., Claes N., Peleman H., Schrooten W. (2012). A nationwide hospital survey on patient safety culture in Belgian hospitals: Setting priorities at the launch of a 5-year patient safety plan. *BMJ Quality & Safety*.

[49] Aboshaiqah A. E., Baker O. G. (2013). Assessment of nurses' perceptions of patient safety culture in a Saudi Arabia Hospital. *Journal of Nursing Care Quality*.

[50] Agnew C., Flin R., Mearns K. (2013). Patient safety climate and worker safety behaviours in acute hospitals in Scotland. *Journal of Safety Research*.

[51] Bahrami M. A., Montazeralfaraj R., Chalak M. (2013). Patient safety culture challenges: Survey results of iranian educational hospitals. *Middle-East Journal of Scientific Research*.

[52] Castañeda-Hidalgo H., Garza Hernández R., González Salinas J. F., Pineda Zúñiga M., Acevedo Porras G., Aguilera Pérez A. (2013). Percepción de la cultura de la seguridad de los pacientes por personal de enfermería. *Ciencia y enfermería*.

[53] Davoodi R., Shabestari M. M., Takbiri A. (2013). Patient safety culture based on medical staff attitudes in Khorasan Razavi Hospitals, northeastern Iran. *Iranian Journal of Public Health*.

[54] Fujita S., Seto K., Ito S. (2013). The characteristics of patient safety culture in Japan, Taiwan and the United States. *BMC Health Services Research*.

[55] Gama Z. A., Oliveira A. C., Hernandez P. J. (2013). Patient safety culture and related factors in a network of Spanish public hospitals. *Cadernos de Saúde Pública*.

[56] Hamdan M., Saleem A. A. (2013). Assessment of patient safety culture in Palestinian public hospitals. *International Journal for Quality in Health Care*.

[57] Kuosmanen A., Tiihonen J., Repo-Tiihonen E., Eronen M., Turunen H. (2013). Patient safety culture in two finnish state-run forensic psychiatric hospitals. *Journal of Forensic Nursing*.

[58] Jones K. J., Skinner A. M., High R., Reiter-Palmon R. (2013). A theory-driven, longitudinal evaluation of the impact of team training on safety culture in 24 hospitals. *BMJ Quality & Safety*.

[59] Moussavi F., Moghri J., Gholizadeh Y. (2013). Assessment of patient safety culture among personnel in the hospitals associated with Islamic Azad University in Tehran in 2013. *Electronic Physician*.

[60] Nie Y., Mao X., Cui H., He S., Li J., Zhang M. (2013). Hospital survey on patient safety culture in China. *BMC Health Services Research*.

[61] Šklebar I., Habek D., Jurković I., Šakić L., Martinac M. (2013). The correlation between patient safety culture and regional anesthesia development. *Periodicum biologorum*.

[62] Turunen H., Partanen P., Kvist T., Miettinen M., Vehviläinen-Julkunen K. (2013). Patient safety culture in acute care: A web-based survey of nurse managers' and registered nurses' views in four Finnish hospitals. *International Journal of Nursing Practice*.

[63] Wagner C., Smits M., Sorra J., Huang C. C. (2013). Assessing patient safety culture in hospitals across countries. *International Journal for Quality in Health Care*.

[64] Wu Y., Fujita S., Seto K. (2013). The impact of nurse working hours on patient safety culture: A cross-national survey including Japan, the United States and Chinese Taiwan using the Hospital Survey on Patient Safety Culture. *BMC Health Services Research*.

[65] Al-Mandhari A., Al-Zakwani I., Al-Kindi M., Tawilah J., Dorvlo A. S. S., Al-Adawi S. (2014). Patient safety culture assessment in Oman. *Oman Medical Journal*.

[66] Bahrami M. A., Chalak M., Montazeralfaraj R., Dehghani Tafti A. (2014). Iranian nurses' perception of patient safety culture. *Iranian Red Crescent Medical Journal*.

[67] Brborović H., Brborović O., Brumen V., Pavleković G., Mustajbegović J. (2014). Are nurse presenteeism and patient safety culture associated: a cross-sectional study. *Archives of Industrial Hygiene and Toxicology*.

[68] El-Jardali F., Sheikh F., Garcia N. A., Jamal D., Abdo A. (2014). Patient safety culture in a large teaching hospital in Riyadh: Baseline assessment, comparative analysis and opportunities for improvement. *BMC Health Services Research*.

[69] Fujita S., Seto K., Kitazawa T., Matsumoto K., Hasegawa T. (2014). Characteristics of unit-level patient safety culture in hospitals in Japan: A cross-sectional study. *BMC Health Services Research*.

[70] Ulrich B., Kear T. (2014). Patient Safety Culture in Nephrology Nurse Practice Settings: Initial Findings. *Nephrology Nursing Journal*.

[71] Wang X., Liu K., You L. (2014). The relationship between patient safety culture and adverse events: A questionnaire survey. *International Journal of Nursing Studies*.

[72] Ammouri A. A., Tailakh A. K., Muliira J. K., Geethakrishnan R., Al Kindi S. N. (2015). Patient safety culture among nurses. *International Nursing Review*.

[73] Bump G. M., Calabria J., Gosman G. (2015). Evaluating the Clinical Learning Environment: Resident and Fellow Perceptions of Patient Safety Culture. *Journal of Graduate Medical Education*.

[74] Farup P. G. (2015). Are measurements of patient safety culture and adverse events valid and reliable? Results from a cross sectional study. *BMC Health Services Research*.

[75] Güneş Ü. Y., Gürlek Ö., Sönmez M. (2016). A survey of the patient safety culture of hospital nurses in Turkey. *Collegian*.

[76] Khater W. A., Akhu-Zaheya L. M., Al-Mahasneh S. I., Khater R. (2015). Nurses' perceptions of patient safety culture in Jordanian hospitals. *International Nursing Review*.

[77] Lawton R., O'Hara J. K., Sheard L. (2015). Can staff and patient perspectives on hospital safety predict harm-free care? An analysis of staff and patient survey data and routinely collected outcomes. *BMJ Quality & Safety*.

[78] Patterson M. E., Bogart M. S., Starr K. R. (2015). Associations between perceived crisis mode work climate and poor information exchange within hospitals. *Journal of Hospital Medicine*.

[79] Raeissi P., Reisi N., Nasiripour A. A. (2015). Assessment of Patient Safety Culture in Iranian Academic Hospitals: Strengths and Weaknesses. *Journal of Patient Safety*.

[80] Saleh A. M., Darawad M. W., Al-Hussami M. (2015). The perception of hospital safety culture and selected outcomes among nurses: An exploratory study. *Nursing & Health Sciences*.

[81] Shu Q., Cai M., Tao H.-B. (2015). What does a hospital survey on patient safety reveal about patient safety culture of surgical units compared with that of other units?. *Medicine (United States)*.

[82] Silva-Batalha E. M. S. D., Melleiro M. M. (2015). Patient safety culture in a teaching hospital: Differences in perception existing in the different scenarios of this institution. *Texto e Contexto Enfermagem*.

[83] Vlayen A., Schrooten W., Wami W. (2015). Variability of Patient Safety Culture in Belgian acute hospitals. *Journal of Patient Safety*.

[84] Azami-Aghdash S., Ebadifard Azar F., Rezapour A., Azami A., Rasi V., Klvany K. (2015). Patient safety culture in hospitals of Iran: A systematic review and meta-analysis. *Medical Journal of The Islamic Republic of Iran*.

[85] Schriesheim C. A., Eisenbach R. J., Hill K. D. (1991). The Effect of Negation and Polar Opposite Item Reversals on Questionnaire Reliability and Validity: An Experimental Investigation. *Educational and Psychological Measurement*.

[86] Blegen M. A., Gearhart S., O'Brien R., Sehgal N. L., Alldredge B. K. (2009). AHRQ's hospital survey on patient safety culture: Psychometric analyses. *Journal of Patient Safety*.

[87] Taylor N., Clay-Williams R., Hogden E., Braithwaite J., Groene O. (2015). High performing hospitals: a qualitative systematic review of associated factors and practical strategies for improvement. *BMC Health Services Research*.

[88] Kringos D. S., Sunol R., Wagner C. (2015). The influence of context on the effectiveness of hospital quality improvement strategies: a review of systematic reviews. *BMC Health Services Research*.

[89] Pronovost P. J., Weast B., Holzmueller C. G. (2003). Evaluation of the culture of safety: Survey of clinicians and managers in an academic medical center. *Quality & Safety in Health Care*.

[90] Makary M. A., Sexton J. B., Freischlag J. A. (2006). Operating room teamwork among physicians and nurses: teamwork in the eye of the beholder. *Journal of the American College of Surgeons*.

[91] Thomas E. J., Sexton J. B., Helmreich R. L. (2003). Discrepant attitudes about teamwork among critical care nurses and physicians. *Critical Care Medicine*.

[92] Olsen E. (2007). MEDISIN OG VITENSKAP-Ansattesoppfatningeravsykehusetssikkerhetskultur. *Tidsskrift for Den norske legeforening*.

[93] Boussat B., François P., Gandon G. (2017). Inconsistencies Between Two Cross-Cultural Adaptations of the Hospital Survey on Patient Safety Culture Into French. *Journal of Patient Safety*.

[94] Centre for Reviews and Dissemination. Systematic Reviews (2009). *CRD’s Guidance for Undertaking Reviews in Health Care: CRD*.

